# Impact of exercise on pulmonary artery pressure in patients with heart failure using an ambulatory pulmonary artery pressure monitor

**DOI:** 10.3389/fcvm.2023.1077365

**Published:** 2023-03-02

**Authors:** Rola Khedraki, Jacob Abraham, Orvar Jonsson, Kunjan Bhatt, Hesham R. Omar, Mosi Bennett, Arvind Bhimaraj, Ashrith Guha, Patrick McCann, Evan D. Muse, Monique Robinson, Andrew J. Sauer, Andrew Cheng, Samantha Bagsic, Marat Fudim, J. Thomas Heywood, Maya Guglin

**Affiliations:** ^1^Division of Cardiovascular Medicine, Scripps Clinic, Prebys Cardiovascular Institute, La Jolla, CA, United States; ^2^Center for Cardiovascular Analytics, Research and Data Science, Providence Heart Institute, Providence Research Network, Portland, OR, United States; ^3^University of South Dakota Sanford Health, Sioux Falls, SD, United States; ^4^Austin Heart, Austin, TX, United States; ^5^Swedish American Hospital, Rockford, IL, United States; ^6^Allina Health Minneapolis Heart Institute, Minneapolis, MN, United States; ^7^Houston Methodist Debakey Heart and Vascular Center, Houston Methodist Hospital, Houston, TX, United States; ^8^PRISMA Health USC Medical Group, Greer, SC, United States; ^9^Scripps Research Translational Institute, La Jolla, CA, United States; ^10^University Hospitals Cleveland Medical Center, Cleveland, OH, United States; ^11^Saint Luke's Mid America Heart Institute, University of Missouri, Kansas City, MO, United States; ^12^Department of Cardiology, Ascension Medical Group, Austin, TX, United States; ^13^Division of Cardiology, Duke University Medical Center, Durham, NC, United States; ^14^Duke Clinical Research Institute, Durham, NC, United States; ^15^Indiana University School of Medicine, Indianapolis, IN, United States

**Keywords:** CardioMEMS, heart failure, hemodynamics, six minute walk test, pulmonary pressures

## Abstract

**Background:**

In this multicenter prospective study, we explored the relationship between pulmonary artery pressure (PAP) at rest and in response to a 6-min walk test (6MWT) in ambulatory patients with heart failure (HF) with an implantable PAP sensor (CardioMEMS, Abbott).

**Methods:**

Between 5/2019 and 2/2021, HF patients with a CardioMEMS sensor were recruited from seven sites. PAP was recorded in the supine and seated position at rest and in the seated position immediately post-exercise.

**Results:**

In our cohort of 66 patients, mean age was 70 ± 12 years, 67% male, left ventricular ejection fraction (LVEF) < 50% in 53%, mean 6MWT distance was 277 ± 95 meters. Resting seated PAPs were 31 ± 15 mmHg (systolic), 13 ± 8 mmHg (diastolic), and 20 ± 11 mmHg (mean). The pressures were lower in the seated rather than the supine position. After 6MWT, the pressures increased to PAP systolic 37 ± 19 mmHg (*p* < 0.0001), diastolic 15 ± 10 mmHg (*p* = 0.006), and mean 24 ± 13 mmHg (*p* < 0.0001). Patients with elevated PAP diastolic at rest (>15 mmHg) demonstrated a greater increase in post-exercise PAP.

**Conclusion:**

The measurement of PAP with CardioMEMS is feasible immediately post-exercise. Despite being well-managed, patients had severely limited functional capacity. We observed a significant increase in PAP with ambulation which was greater in patients with higher baseline pressures.

## Introduction

Exercise intolerance is a cardinal manifestation of heart failure (HF). The standard 6-min walk test (6MWT) quantifies functional capacity and correlates strongly with the risk of heart failure (HF) hospitalization and mortality in patients with HF, regardless of ejection fraction (EF) (1–3). Hemodynamic parameters measured at rest and with activity have shown modest correlation to 6MWT distance in chronic heart failure patients when measured invasively in a supine position. Elevation of filling pressures is the strongest predictor of 6MWT distance, and importantly, is correlated to survival in HF. Taken together, these findings suggest that lowering of filling pressures can improve functional capacity and clinical outcomes in HF.

Heart failure management guided by an implantable pulmonary artery pressure sensor (CardioMEMS®, Abbott, IL, United States) reduces pulmonary artery pressure (PAP) and HF hospitalizations in HF patients compared to standard-of-care HF management as was demonstrated in the landmark CHAMPION trial (4). Ambulatory hemodynamic management may also reduce HF mortality (5). More recently, in a pre-pandemic analysis, the GUIDE-HF trial showed a statistically significant and favorable reduction of the primary end point (all-cause mortality, HF hospitalizations, urgent heart failure visits) which was largely driven by a reduction in heart failure hospitalizations (6). Based on the results of this trial, the United States Food and Drug Administration (FDA) granted approval for an expanded indication of CardioMEMS HF system to include earlier-stage HF patients.^1^

We hypothesized that PAP measured using an implantable sensor after a 6MWT in both supine and seated hemodynamic position could allow for more accurate correlation of hemodynamic variables with functional capacity. We therefore conducted a prospective multi-center pilot study to examine the effect of 6MWT on pulmonary pressures using the CardioMEMS (CM) sensor in patients with chronic HF.

## Methods

Patients who had a CM sensor implanted for clinical indications were recruited from seven sites across the United States from May 2019 to February 2021. PAP was recorded from the CM in the supine and seated position at rest and in the seated position post-exercise.

Non-invasive blood pressure (BP) and heart rate (HR) were measured immediately pre- and post-exercise during PAP measurement from the CM with the patient in an upright seated position. Pulmonary artery systolic pressure (PASP), diastolic pressure (PADP) and mean pressure (PAMP) were recorded immediately before and after exercise. Upon completion of exercise the patient was seated with goal of measuring CM pressures within 30 s after termination of the 6MWT.

Demographic data including New York Heart Association (NYHA) class, HF etiology (i.e., ischemic vs. non-ischemic), LVEF, comorbidities, cardiac medications, and implantable cardiac electronic device settings including rate-response information was collected. PAP data was measured at end expiration and PAP tracings from all sites were sent in de-identified form to Scripps Clinic for central adjudication by a heart failure cardiologist.

Baseline characteristics were summarized as means and standard deviations for continuous variables and as frequencies and percentages for categorical variables. Pre-and post-exercise data were compared with a paired *t*-test. Difference among the groups was compared with a one-way ANOVA test.

## Results

Sixty-six patients with a mean age of 70 ± 12 were enrolled. The majority were male (67%), NYHA class III (61%), with an LVEF <50% (53%). Five patients were NYHA I (7.6%), 17 NYHA II (25.8%), 40 NYHA III (60.6%), and 4 NYHA IV (6.0%) (Table 1). There were no differences between patients with normal and reduced LVEF, except for greater treatment with sacubitril/valsartan and implantable devices in patients with reduced LVEF.

**Table 1:** Baseline Characteristics of patient cohort.

	Total		LVEF ≥ 50% (*N* = 31)		LVEF < 50% (*N* = 35)		
*N* = 66	Mean	SD	Mean	SD	Mean	SD	*p*-Value
Age, years	70.0	11.7	72.1	7.2	68.11	14.4	0.16
Body mass index	32.0	7.1	31.37	7.6	32.47	6.7	0.54
LVEF, %	44.8	13.6	56.43	5.4	34.45	9.6	<0.0001
*N* = 66	*n*	%	*n*	%	*n*	%	*p*-value
Female	22	33.3%	12	38.7%	10	28.6%	0.44
Race							0.78
Caucasian	60	90.9%	29	93.6%	31	88.6%	
Black	2	3.0%	0	0.0%	2	5.7%	
Asian	4	6.1%	2	6.5%	2	5.7%	
NYHA class							0.82
I	5	7.6%	3	9.7%	2	5.7%	
II	17	25.8%	9	29.0%	8	22.9%	
III	40	60.6%	17	54.8%	23	65.7%	
IV	4	6.1%	2	6.5%	2	5.7%	
Hypertension	54	81.8%	27	87.1%	27	77.1%	0.35
Coronary artery disease	37	56.1%	16	51.6%	21	60.0%	0.62
Diabetes	30	45.5%	17	54.8%	13	37.1%	0.22
Pacemakers/ICDs							<0.0001
None	31	47.0%	21	67.7%	10	28.6%	
Single lead	20	30.3%	10	32.3%	10	28.6%	
Dual chamber	11	16.7%	0	0.0%	11	31.4%	
Biventricular	4	6.1%	0	0.0%	4	11.4%	
Beta blockers	60	90.9%	26	83.9%	34	97.1%	0.09
ACE/ARB	13	19.7%	7	22.6%	6	17.1%	0.76
Aldactone	27	40.9%	9	29.0%	18	51.4%	0.08
Loop diuretics	61	92.4%	27	87.1%	34	97.1%	0.18
Thiazides	5	7.6%	0	0.0%	5	14.3%	0.06
Calcium channel blockers	7	10.6%	6	19.4%	1	2.9%	0.05
Nitrates	17	25.8%	8	25.8%	9	25.7%	1.00
Sacubitril/valsartan	17	25.8%	1	3.2%	16	45.7%	<0.0001
Statin	41	62.1%	17	54.8%	24	68.6%	0.31

ICD, implantable cardioverter-defibrillator; LVEF, left ventricular ejection fraction; NYHA, New York Heart Association.

**Figure 1:**
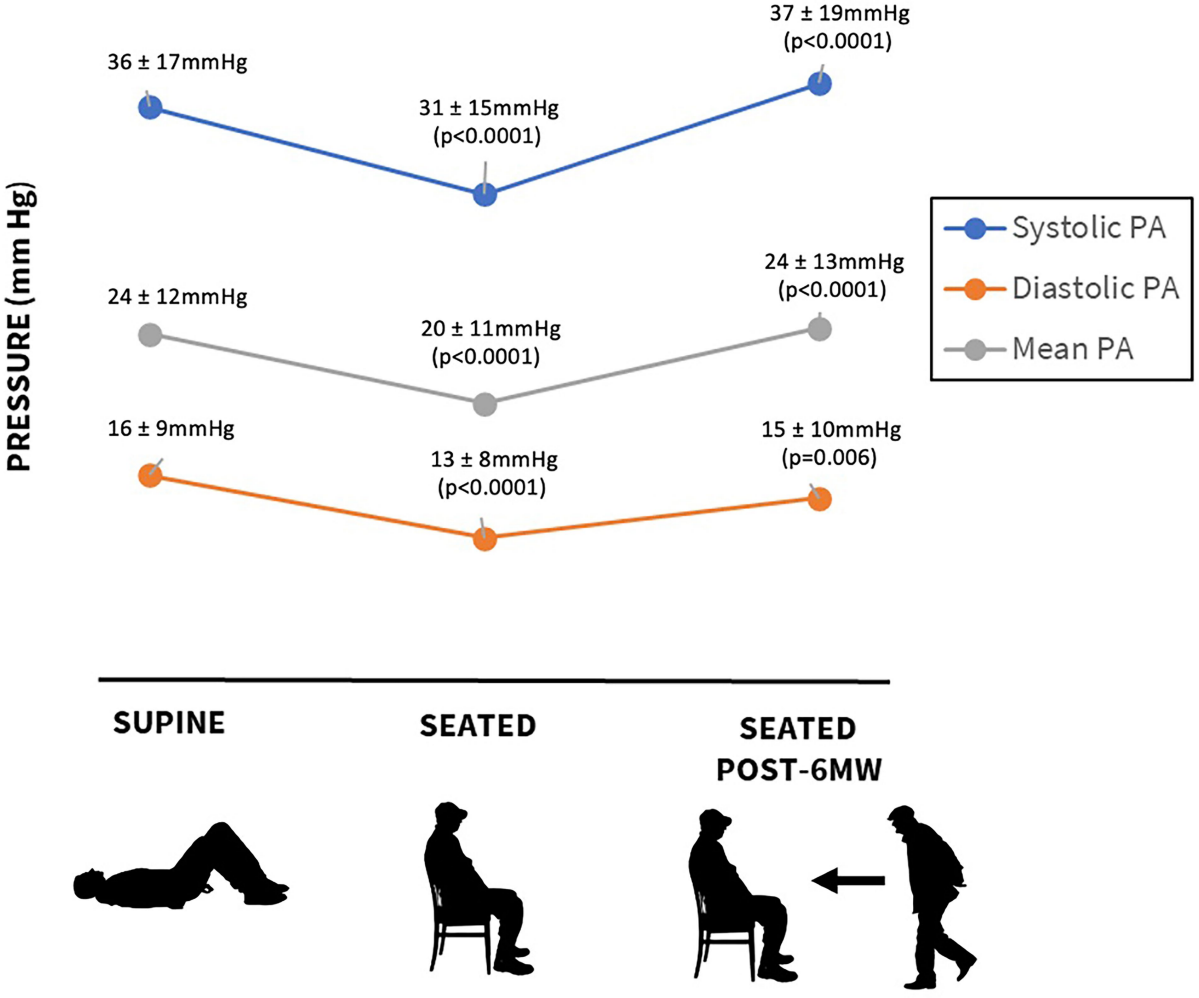
Changes in pulmonary pressures based on position and post 6-min walk (6MW).

The mean 6MWT distance was 277 ± 95 m. Resting supine pressures were PASP 36 ± 17, PADP 16 ± 9, and PAMP 24 ± 12 mmHg (Figure 1). In the seated position, the corresponding values were lower at 31 ± 15, 13 ± 8, and 20 ± 11 mmHg (*p* < 0.0001). Compared to pre-exercise seated pressure, pressures measured after 6MWT increased: PASP 37 ± 19 mmHg (*p* < 0.0001), PADP 15 ± 10 mmHg (*p* = 0.006), and PAMP 24 ± 13 mmHg (*p* < 0.0001). Mean increases after exercise were PASP 6 ± 11, PADP 2 ± 6, and PAMP 4 ± 8 mmHg. There was no difference in 6MWT distance or pre-and post-exercise pulmonary pressures in patients with normal versus reduced LVEF. Resting HR in the seated position was 76 ± 13 beats per minute, and it increased to 86 ± 15 beats per minute after the walk, *p* < 0.001.

In 46 patients (70%), resting supine PADP was <20 mmHg. Only 28 patients (42%) were able to walk 300 m or more. There was a negative correlation of 6MWT distance with NYHA class, PASP, PADP, and PAMP supine, and PASP seated (*r* = −0.28, –0.31, –0.27, –0.31, and −0.27, respectively, all *p* < 0.01). The distance walked in 6 min decreased significantly with increasing NYHA functional class (NYHA I to IV: 287 ± 98, 335 ± 96, 255 ± 87, and 241 ± 92 m, respectively, *p* = 0.026). There were no sex-related differences in any pre-or post- exercise parameters.

**Figure 2:**
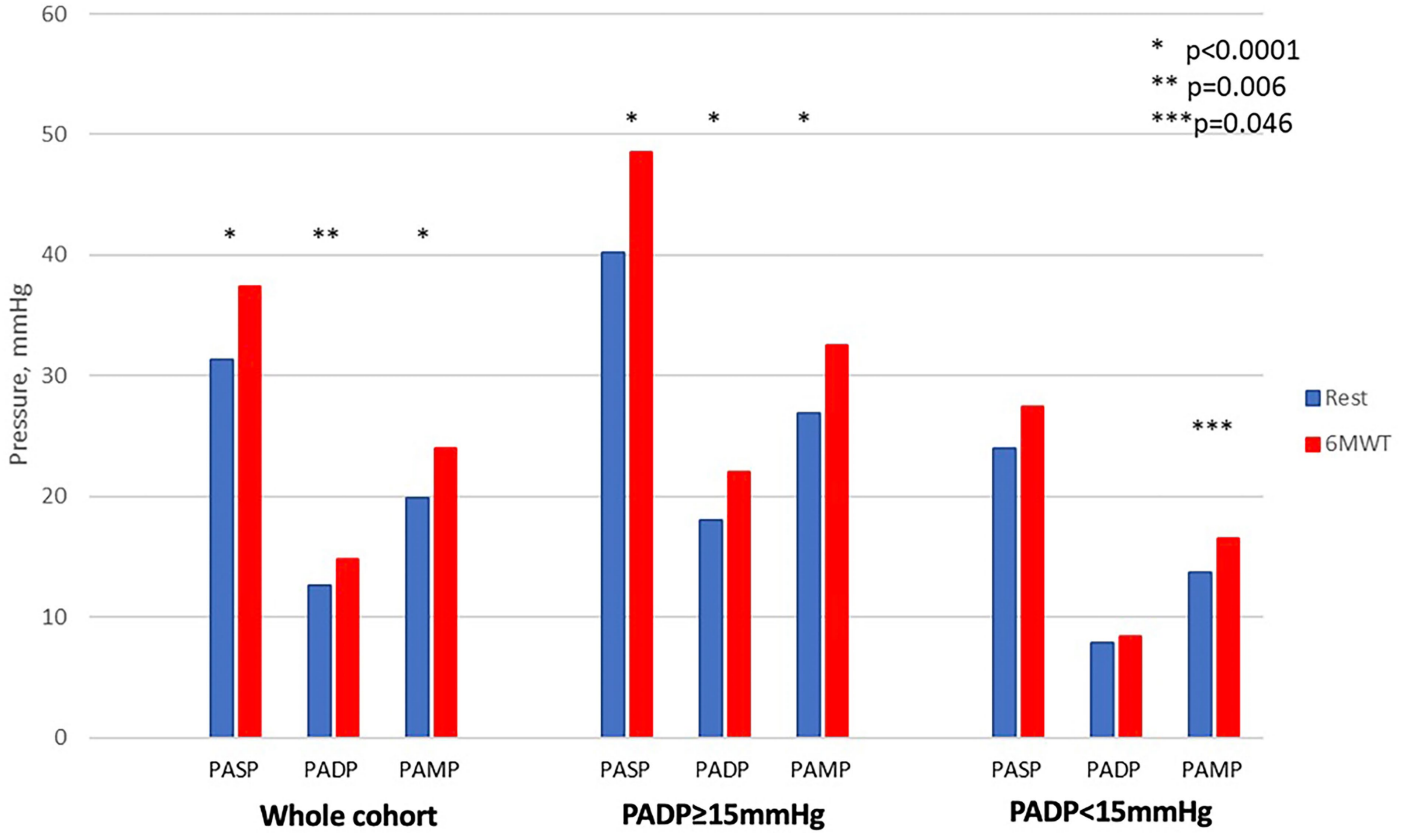
Changes in pulmonary pressures from resting supine to post-exercise in the whole cohort and in subgroups with elevated and normal pulmonary artery diastolic pressure in the supine position.

As resting PADP is a therapeutic target in hemodynamic management with CM, we further stratified the cohort according to resting pressures. Because PADP is usually measured in a supine position in clinical practice, we used supine resting PADP to define cohorts with PADP≥15 mmHg and <15 mmHg. After 6MWT, there was a significant increase in all pulmonary pressures in patients with PADP ≥15 mm Hg (*p* < 0.001) but not in individuals with PADP <15 mm Hg (Table 2 and Figure 2). Heart rate increased significantly in both groups: from 78 ± 12 to 86 ± 14 beats per minute (*p* = 0.004) and from 74 ± 13 to 86 ± 16 beats per minute (*p* < 0.001), respectively. There was no correlation between PADP and 6MWT distance.

**Table 2:** Changes in pulmonary pressures from rest to post-exercise in the whole cohort and in subgroups with elevated and normal pulmonary artery diastolic pressure in the supine position.

	Rest (Supine)		6MWT		
	Mean	SD	Mean	SD	*p*-Value
Total (*N* = 66)					
PASP, mmHg	31.3	14.9	37.4	18.7	<0.0001
PADP, mmHg	12.6	8.4	14.8	9.6	0.006
PAMP, mmHg	19.9	10.6	24.0	13.1	<0.0001
PADP ≥ 15 mmHg (*N* = 31)					
PASP, mmHg	40.3	15.1	48.6	19.7	<0.001
PADP, mmHg	18.1	6.4	22.0	8.1	<0.001
PAMP, mmHg	26.9	9.4	32.5	12.7	<0.001
PADP < 15 mmHg (*N* = 35)					
PASP, mmHg	23.9	9.7	27.4	10.5	0.078
PADP, mmHg	7.8	6.9	8.4	5.2	0.638
PAMP, mmHg	13.7	7.2	16.5	7.7	0.046

6MWT, 6-min walk test; PADP, pulmonary arterial diastolic pressure; PAMP, pulmonary arterial mean pressure; PASP, pulmonary arterial systolic pressure; SD, standard deviation.

## Discussion

The major findings of this study are: (1) measurement of pulmonary artery pressure after exercise was feasible in all patients; (2) pulmonary artery pressures fall significantly with change from supine to seated position, with the predominant effect on PASP; (3) with 6MWT, PAP increased significantly, regardless of EF; (4) the strongest hemodynamic correlate of 6MWT distance was supine PAP.

Our study population represents a unique cohort of patients with chronic HF treated with the guidance of an implantable hemodynamic sensor (CardioMEMS). Despite being well managed, with 70% having PADP <20 mmHg at rest, which represents a typical hemodynamic goal (7), the cohort was noted to be exceptionally limited with physical activity. Less than 50% were capable of covering a distance of 300 m or more during a 6MWT, and the mean distance was 277 ± 95 m. Although a self-paced 6MWT represents mild exercise, comparable with activities of daily living, the patients experienced a significant increase in pulmonary pressures after walking regardless of preserved or reduced LVEF. Pulmonary pressures in our cohort were higher not only after exercise, but also in the supine position compared with the upright seated position likely due to gravitational influences. Interestingly, the 6MWT distance demonstrated a consistent negative correlation with supine but not upright or post-exercise pulmonary pressures. We also observed a significant chronotropic incompetence in this cohort which likely led to functionally limitation during 6MWT.

CardioMEMS provides instantaneous values for PASP and PADP in ambulatory individuals. The calibration of the sensor occurs during the implantation, and if no major discrepancy is identified, PADP is later used as a surrogate of pulmonary capillary wedge pressure. Typically, the target value for HF patients is set at PADP below 20 mmHg (7). The hemodynamic response to exercise in patients with HF has been assessed most commonly during maximal exercise stress tests (8). Typically, the heart rate and all pulmonary pressures increase while mixed venous oxygen saturation decreases. In healthy individuals, pulmonary capillary wedge pressure increases during exercise, reaching up to 20 mmHg (9). In a significant proportion of our patients, PAP remained elevated even at rest despite treatment with guideline directed medical therapy (GDMT) and with targeted diuretic treatment to reduce or maintain PADP goals, underscoring that pulmonary hypertension in HF may be incompletely treated or only partially reversible (10). Elevation of pulmonary pressures out of proportion to the rise in cardiac output with exercise is considered a key mechanism underlying symptoms such as exertional dyspnea. In patients who have normal resting hemodynamics, exercise can unmask increases in pulmonary capillary wedge pressure that indicate underlying heart failure (11).

While there is ample evidence that higher pulmonary pressures are associated with morbidity and mortality in HF (12), the response of pulmonary pressures to daily activities such as walking is less studied. In the studies by Gibbs et al. (13) using an invasive pulmonary artery catheter, PASP in patients with HF reached 59.4 ± 26.1 mmHg with treadmill exercise, 54.9 ± 30.6 mmHg with bicycle exercise, 52.5 ± 26.1 mmHg walking stairs and 43.5 ± 23.9 mmHg walking on a flat surface. Corresponding values for PADP were 27.8 ± 14.6, 26.5 ± 14.9, 24.9 ± 14.8, and 26.4 ± 12.5 mmHg, respectively (13). Although this study similarly evaluated pulmonary pressures with ambulation on flat surfaces, we observed lower PAPs. This difference likely reflects both the longer exercise time in the Gibbs study (10 min) as well as contemporary, hemodynamic-guided HF management of our patient cohort.

We found that patients with higher baseline PADP experienced a more pronounced increase in pulmonary pressures with exercise. Although this increase in pressure did not impact 6MWT distance, the inverse correlation of 6MWT distance with supine pulmonary pressures shows that the efforts to decrease PAP in ambulatory patients with HF remains a worthy goal.

This study has several important limitations. First, the 6MWT is a self-paced test that is submaximal and less rigorous than other exercise protocols or modalities. Distance walked is impacted by multiple non-cardiac variables including age, sex, nutritional status, cognitive status, peripheral artery disease, weight, etc. (14–16). However, the advantages of 6MWT are its ease of performance and reproducibility. A second limitation is the inability to measure cardiac output (CO) simultaneously with PAP, limiting our ability to ideally measure changes in pressure indexed to changes in flow (i.e., ∂PAP/∂CO). Third, the limited functional capacity of the cohort could be related to suboptimal medical management of patients with reduced EF. Although generally well treated with 97% of patients with LVEF <50% on beta blockade, only 51% were on aldosterone antagonist therapy and 45% on ARNI therapy. The reasons for decreased GDMT uptake were not recorded. Interestingly, a sizeable proportion of the LVEF>50% cohort were treated with beta blockers (84%), which may have also played a role in the chronotropic incompetence observed.

## Conclusion

In this cohort of ambulatory patients with HF managed with an implantable pulmonary artery pressure sensor, patients exhibited limited functional capacity with a mean 6MWT distance of 277 ± 95 m. PAP were higher in the supine than the seated position. Measurement of pulmonary artery pressures post-exercise with the CardioMEMS system was feasible. We observed a significantly increase in PAP with walking. The increase was greater in patients whose rest PADP exceeded 15 mmHg. Further studies should explore optimal management strategies in this patient population to improve hemodynamics and functional capacity.

## Summary

- CardioMEMS is a sensor that has been shown to assist in guiding the management of patients with heart failure remotely by monitoring pressures in the pulmonary artery.
- Usually the pressures are measured while patients are laying down (supine).
- This study examined the ability to use a CardioMEMS sensor to monitor the effect of positional changes (supine vs. seated) and exercise on pulmonary pressures.
- We found that pulmonary pressures increase in the supine position and after exercise and that the greatest increase was seen in patients with baseline elevated pressures. Furthermore, supine pressures most reliably predicted walk distance.

## Data availability statement

The raw data supporting the conclusions of this article will be made available by the authors, without undue reservation.

## Ethics statement

The studies involving human participants were reviewed and approved by Institutional Review Board. The patients/participants provided their written informed consent to participate in this study.

## Author contributions

RK, JA, and JH: writing and data collection. OJ, KB, MB, AB, AG, PM, MR, and AC: data collection. HO and SB: statistical analysis. EM, AS, and MF: writing. MG: writing, data collection, and statistical analysis. All authors contributed to the article and approved the submitted version.

## Conflict of interest

JA, OJ, MF, and KB are speakers for Abbott. KB also serves on the speakers bureau for Pfizer and Novartis and serves as a consultant for Abbott and Vectorius. MB and AB serve as consultants for Abbott. PM serves as a consultant for Abbott, AstraZeneca, Janssen, Daxor, and CVRx. AS reports receiving research funding from Abbott as part of the GUIDE-HF trial and reports serving on steering committees for Abbott, Boston Scientific, Biotronik, Story Health, and General Prognostics. AS also reports receiving consulting honoraria from Abbott, Boston Scientific, Edwards Lifesciences, Biotronik, Story Health, and General Prognostics. JH has received honoraria from Actelion Pharmaceuticals, Medtronic, Abbott, Boehringer, Ingelheim, Bayer, Lily as well as research grant support from Abbott and Impedimed.

The remaining authors declare that the research was conducted in the absence of any commercial or financial relationships that could be construed as a potential conflict of interest.

## Publisher’s note

All claims expressed in this article are solely those of the authors and do not necessarily represent those of their affiliated organizations, or those of the publisher, the editors and the reviewers. Any product that may be evaluated in this article, or claim that may be made by its manufacturer, is not guaranteed or endorsed by the publisher.

## Footnotes

^1^ https://www.fda.gov/medical-devices/recently-approved-devices/cardiomems-hf-system-p100045s056
